# What difficulties do faculty members face when conducting workplace-based assessments in undergraduate clerkships?

**DOI:** 10.5116/ijme.5689.3c7f

**Published:** 2016-01-23

**Authors:** Hester E.M. Daelmans, Marianne C. Mak-van der Vossen, Gerda Croiset, Rashmi A. Kusurkar

**Affiliations:** 1 VUmc School of Medical Sciences, Institute of Education and Training, Amsterdam, the Netherlands

**Keywords:** Mini-CEX, workplace-based assessment, faculty development

## Abstract

**Objectives:**

Workplace-based assessments are based on the principle of providing feedback to medical students on clinical performance in authentic settings. In practice, however, the assessment often overshadows the feedback. The aim of this study was to determine what problems faculty perceived when performing workplace-based assessments and what solutions they suggested to overcome these difficulties.

**Methods:**

Discussion meetings were conducted with education coordinators and faculty (n=55) from 11 peripheral hospitals concerning the difficulties encountered when conducting workplace-based assessments. We analysed the reports from these discussion meetings using an integrated approach guided by our research questions to code the data. Two researchers analysed the data independently and resolved differences of opinion through consensus.

**Results:**

The problems perceived by faculty in workplace-based assessments (difficulties) and suggestions for improvement formed the overarching themes. Problems included the short duration of clerkships, students choosing the assessment moments, the use of grades for the mini-Clinical Evaluation Exercise, the difficulty in combining teacher and assessor roles and the difficulty in giving fail judgements. Suggestions for improvement included longer clerkship duration, faculty choosing the assessment moments, using a pass/fail system for the mini-Clinical Evaluation Exercise and forward feeding of performance from earlier clerkships following a fail judgement.

**Conclusions:**

Our study indicates that faculty perceive difficulties when conducting workplace-based assessments. These assessments need periodical review to understand the difficulties faculty experience using them; they also require periodical feedback to ensure their proper and effective use.

## Introduction

Workplace-based assessments (WBAs) are meant to provide feedback to medical students and residents on their performance in authentic settings during actual interaction with patients.^1^ Several forms of assessment can be used to perform WBAs, such as case-based discussions, use of multisource feedback and portfolios, the Clinical Evaluation Exercise and the mini-Clinical Evaluation Exercise (mini-CEX).^1^^,^^2^ The mini-CEX is meant to be carried out by different supervisors at different points in time to provide students with immediate feedback on their clinical performance. It assesses the competence of students in history taking, physical examination, professionalism, clinical judgement, communication skills, organizational skills and overall clinical care.^3^ The mini-CEX is intended to be used as a formative assessment, but it also has a small summative component that contributes to the final grade.^3^ These principles of the mini-CEX should ideally enable feedback on performance.^4^ However, in spite of the published descriptions of best practices in formative WBAs, there is a vast discrepancy between theory and practice. The following reasons for clinician-educators failing to provide effective feedback have been suggested: assessments such as the mini-CEX place more emphasis on the assessment than on the feedback, educators lack the skills for providing feedback and scoring sheets include insufficient space for feedback.^5^

At the VUmc School of Medical Sciences, students undertake their clerkships in the university hospital, which is a tertiary care hospital, as well as in other peripheral hospitals (≤20). Faculty conduct WBAs several times during each clerkship, mainly using the mini-CEX, which was introduced in 2010. Throughout their clerkships, students carry evaluation booklets which include a section with forms for the mini-CEX, and they have to complete a minimum number of mini-CEX assessments. When faculty do a mini-CEX, they complete the students’ evaluation form. Faculty are offered specific assessment training to perform mini-CEX and to give feedback based on the mini-CEX and WBAs. Although participation in this training is voluntary, the majority of the faculty have taken up the offer.

Each mini-CEX can be considered a single assessment data point that contributes to the summative result: the final score on the WBA.^6^ Together with the performance on mini-CEXs, the summative WBA score takes into account professional behaviour. Professional behaviour is taught and assessed in a longitudinal manner throughout our medical programme,^7^ where feedback on professional behaviour provided in the WBA is considered important for learning such behaviour and for remedial action in the case of fail judgements.^8^ Van Luijk et al.  have defined professionalism as ‘Having specialized knowledge and skills, acquired through extensive study, training and experience, being able to apply this within the rules that have been drafted by the profession itself, the organization and the government, in which one can be held accountable for actions by all parties involved. This needs to be placed within the cultural context and time frame in which the term is used’.^9^ Professional behaviour has further been defined as ‘The observable aspects of practicing professionalism’. We have translated this definition into practical and observable skills, and we have described them as a tool for assessing professionalism.^10^ Thus, in our setting, professional behaviour is defined as having the skills to 1) deal with tasks, 2) deal with others and 3) deal with oneself.

The literature on WBAs suggests that in practice there is too much focus on assessment and that feedback tends to be neglected. We therefore wanted to gain insight into the problems our clinician-educators face when performing WBAs; we also wanted to know whether faculty had any solutions for those problems that could be adopted by other medical schools. In 2013, we therefore organised discussion meetings with the education coordinators and faculty at the peripheral hospitals with the aim of improving our clerkship programme. During these meetings, we specifically gathered information on how the faculty perceived WBAs. The aim of the study was to determine what problems faculty faced in performing WBAs and what solutions they could suggest to overcome these difficulties. This paper is a report on our findings from those meetings.

## Methods

### Study design

The study used a constructivist paradigm, which sees knowledge as constructed through interaction between participants and researchers.^11^ Following the constructivist paradigm the researchers (HD and RAK) were actively involved in the discussion sessions held with purposefully chosen participants. We reflected on our roles in the research process in research team discussions.

In order to allow readers to make a meaningful interpretation of our work, we share the following information about the researchers’ backgrounds with the readers: the first author (HD) is a medical doctor, head of the Master programme and an expert in assessment during clerkships. The co-authors are also medical doctors with different responsibilities and experiences within the educational organization, including membership of the Examination Board (RAK) and coordination of the educational domain of professional behaviour (MM). All are researchers in medical education (HD, GC, RAK, MM).

### Setting

The Vrije University Medical Center (VUmc) School of Medical Sciences has a competency-based curriculum that follows the Bachelor/Master structure. Three years of preclinical education are followed by a 3-year master of medicine, consisting mainly of clerkships in the major clinical disciplines, as well as in public health, nursing home care and family medicine. The duration of clerkships varies from 2 to 16 weeks. In these clerkships, we used mini-CEX^12^ for feedback on and assessment of clinical consultation skills. Students’ performance in writing patient reports and their more ‘hands on’ performance were observed and assessed using specific forms. The students collected both written feedback and grades in a specially designed evaluation booklet. A formative midterm evaluation was followed by a summative end-of-term evaluation, which resulted in a grade. These evaluations (which we call the WBA) included both the judgements provided in the evaluation forms in the booklet and the global judgements made by faculty concerning professional behaviour. Overall, these evaluations lead to a single global judgement on clinical performance and professional behaviour.

### Sample collection and size

In the period June–December 2013 we held discussion meetings in 11 peripheral hospitals, in which ≥3 disciplines offered clerkships to our students. Faculty members and education coordinators (n=55, 2–11 per hospital) participated in these meetings. Hereinafter, we simply refer to them as ‘faculty’.

### Participants

The educational coordinator of the particular hospital, a number of faculty members of the particular hospital (on average six) and four members of the team of the medical school participated in the discussion meetings.

We visited the hospitals for two purposes: first, to collect information on the experiences of faculty to improve our clerkship programme; second, to serve as a platform for discussing issues faculty encountered during WBAs. Based on these two aims, we chose discussion meetings as a means of collecting data. The team of the medical school consisted of HD, RAK, one educational coordinator from our medical school and a secretary to record the minutes of the meetings.

We consulted the faculty prior to the meetings to draw up the agenda for their hospital. The proposed agendas of the hospital faculty and the medical school were combined to formulate the agenda for every meeting, which served as a discussion guide. All the agendas included the following items concerning WBAs: observation of and feedback to medical students, assessment and WBA experiences, difficulties with and how to improve on the WBAs, the extent of student-patient encounters in the clerkships and faculty development in performing WBAs.

After every meeting, the team of the medical school discussed the findings and honed the agenda for the following meeting in a different hospital. Because the hospital staff contributed their own points, we considered the likelihood of not obtaining new data to be low. The agenda served only as a guide, and the format of the discussion during the meetings was very open. There was ample space to elaborate on every topic as well as to bring in new topics.

### Data collection

The meetings were all chaired by HD. During the meeting (duration 2 hours), we addressed experiences with mini-CEXs and WBAs, including professional behaviour assessment. Detailed minutes were taken and every meeting resulted in a report and a to-do list. All reports were handled anonymously and were finalized immediately after a member check and approval from all participants.

### Data analysis

HD and RAK independently analysed the reports using an integrated approach guided by the research questions to code the data. They searched for codes describing difficulties faced in WBAs and possible solutions to enable better implementation of the mini-CEX. This resulted in a coding scheme that was discussed in the research team in terms of its accuracy. Any differences of opinion were resolved through consensus. The findings were discussed with the full research team, which led to critical appraisal by the rest of the team. The themes and sub-themes were finalized by the full research team.

Ethical approval for this study was obtained from the Dutch Medical Education Association – Ethical Review Board (NVMO-ERB, file no. 462).

## Results

The aims of the discussion meetings, and the overarching themes in our analysis, were to identify the difficulties experienced by faculty in WBAs and any possible solutions they saw.

### Difficulties experienced by faculty

#### Clerkships are too short to make a fair and reliable judgement

The faculty reported difficulty in forming judgements, attributed to having ‘too little’ time to observe the students in the relatively short clerkships: ‘Some clerkships are so short that I don’t have enough time to observe the student to judge him.’

#### Students determine the evaluation moments

The faculty noticed that students determine the moments of their mini-CEX, selecting those when they gave their best performance and thus receiving high grades: ‘When after a consultation I tell the student it was a good performance, the student hands over the booklet knowing that the mini-CEX will be graded as good.’

#### Problems with grades

The faculty were unhappy with the use of grades in the mini-CEX because they perceived that students were interested only in obtaining high grades and did not pay appropriate attention to the feedback that was provided: ‘The students expect a high grade and if they do not get this, they only discuss why they did not get a high grade.’

#### Insufficient space for feedback

The faculty found that the space provided in the mini-CEX evaluation form was insufficient to give good narrative feedback to the student: ‘There is too little space in the judgement form to give good narrative feedback.’

#### Difficulty in combining roles

The faculty found it difficult to combine the roles of a supervisor and an examiner because in their opinion they were then evaluating their own teaching. These roles can thus be conflicting in terms of their own interests: ‘When I judge the student, I essentially judge my own teaching.’

#### Barriers to giving fail judgements for clerkships

The faculty perceived giving fail judgements in clerkships as ‘creating extra work’ for themselves as they were required to provide additional information on why and how this judgement was reached. Thus, on top of their time invested in teaching and supervision, they had to make extra time for the effort involved in giving a fail judgement: ‘Giving a fail judgement means extra work. You have to talk to the student and this is a lot of work, especially when the student does not agree with you. Then the medical school comes in and asks for extra justification for the fail on top of the reasons I have already given on the form. It sometimes feels as if the medical school does not support my judgement.’

The faculty also sometimes felt insecure and stressed about giving a fail judgement because they felt they had observed the student too little and at other times because they had missed information on below-average performance in earlier clerkships. In short, they reported they would feel much better if they had reassurance from colleagues concerning the ‘fail’ judgement they had given.

### Possible solutions to overcome the difficulties with WBAs

#### Faculty should determine the evaluation moments

To make fair and reliable judgements, the faculty felt that ‘when’ and ‘by whom’ the evaluation (for mini-CEX) happens should be chosen by them and not by the students.

#### Movement of the grading system to a feedback-rich system with only pass/fail judgments

Faculty were of the opinion that the students were only interested in grades, which distracted from improving their competencies. Faculty therefore pleaded for the use of feedback combined with a pass/fail system instead of grades on the mini-CEX: ‘The students expect a high grade and if they do not get this, they only discuss why they did not get a high grade. I find that feedback on the performance is far more important than a discussion of the grade itself.’

#### Creating better ways for students to receive supervision

Some ways of ensuring better supervision for the students were suggested by the faculty. One suggestion was to have the students generate a personal development plan at the beginning of each clerkship, to outline learning objectives, and share it with the faculty. Should the student not offer to do this voluntarily, the teacher could actively ask for such a plan. Another solution was to have an intake interview with the student to gather information on performance in earlier clerkships. This could be followed up with the student and faculty filling in the evaluation form for the student simultaneously and separately and then comparing notes. Any discrepancies in opinions could be used to set up new learning objectives: ‘I ask my students to make a personal development plan at the beginning of the clerkship and share it with me.’

#### Forward feeding of information on student competence

Faculty felt this would help in judging and supervising students more effectively, as it could help tailor their supervision to the student’s needs. When they heard that forward feeding of information was against the policy of the medical school, which is designed to protect the privacy of students, they questioned this thinking. They argued that forwarding information happens regularly for residents, and they wondered why it was not possible to receive information on an undergraduate student’s performance in earlier clerkships: ‘Why is it not possible to forward the feedback to the next clerkship? You get a better impression of the student and the student can benefit from it! I think the privacy issue is too restrictive in this; in residency training it is possible to do forward feeding.’

#### Feedback about the progress of a student after giving a fail judgement

The faculty would like updates on the progress of a student who has been awarded an unsatisfactory professional behaviour judgement by them. They felt insecure about their judgment (‘Have I identified it correctly?’) and unsupported by the medical school when they did not hear what happened to the student later. They felt a need to have a longitudinal follow-up system for each student, which would make it easier to feed forward information on performance from one clerkship to the next: ‘After giving a fail judgement, I would like to know what actions the medical school takes and the progress the student made after the judgement.’

## Discussion

We wanted to determine what problems faculty perceived when performing workplace-based assessments and what solutions they suggested to overcome these difficulties. Faculty thought that some clerkships were too short to provide a fair and reliable evaluation of student’s competencies because they experienced few opportunities to observe the student. The opinions of the faculty on what they considered ‘too short’ for a clerkship ranged from 2 to 4 weeks. This raises the question of what the ideal minimal duration of a clerkship is for faculty to be able to provide fair and reliable judgements, which is in turn related to the question of how many times faculty should observe a student to give fair and reliable judgements.

In the literature, 10–12 encounters have been recommended for the mini-CEX to achieve a reproducibility of 0.80.^12^ Fewer encounters can be sufficient for a student whose performance is very high or very low, but more than 10 encounters are needed if the student’s performance is borderline or below average.^12^^,^^13^ It thus becomes quite clear that a 2–4 week clerkship is not sufficient to carry out the recommended number of patient encounters.

Faculty were of the opinion that students’ workplace learning was mainly led by the mini-CEX and that students placed far too much stress on grades. They saw that students therefore approached these assessments in a very strategic manner. They looked out for faculty to perform assessments and get them recorded in their evaluation booklets at opportune moments, i.e. after having performed well in the clinic or on a particular case, or when the supervisor was likely to be lenient or would have little time to evaluate their performance critically.

The tenet that ‘assessment drives learning’ is well known in medical education.^14^^,^^15^^,^^16^  We think we can make better use of this phenomenon by suspending the use of grades for the mini-CEX to divert students’ focus from grades to improving their competencies through the narrative feedback they receive. Another solution proposed by the faculty was to have the supervisors choose the moments for evaluation. In addition, the mini-CEX forms should provide sufficient space to give faculty the opportunity to provide feedback to students in a narrative way, bearing in mind the essential components for effective feedback.^17^^,^^18^ This finding has also been reported in the literature.^5^

Faculty perceived combining their roles as supervisor and assessor for the same students as an internal conflict. They felt that they might not be critical enough in making judgements because they were at least partly evaluating their own teaching. A different, but related, problem has previously been reported in postgraduate training: faculty established a personal connection with the trainees, which made it difficult for them to give fail judgements.^19^ These are two different problems which raise the same question: ‘How we can segregate the roles of a supervisor and an assessor in educational practice?’

To our knowledge, this finding has not previously been reported in undergraduate medical education. For postgraduate education, Driessen and Scheele^19^ do recommend shifting the emphasis from assessment of trainee performance to learning of the trainee and suggest a portfolio system for providing greater reliability of judgements during postgraduate education. Introducing a portfolio system with a focus on learning could also be a solution for undergraduate medical education. With the focus on learning, it is likely that the faculty will not perceive it as ‘evaluating their own teaching’.

Giving a fail judgement in WBAs, especially for professional behaviour, was experienced as personally ‘difficult’ by the faculty. In the literature, this is described as a ‘reluctance to fail’.^20^^,^^21^ They hoped for reassurance from colleagues based on fail judgements awarded to the student in earlier clerkships. As our system does not permit faculty to be provided with information on students’ clinical and professional performance in earlier clerkships, the faculty actively requested introducing this as a routine procedure. Currently we do not have a solution for this problem. Some faculty suggested that they would like to be involved in formulating the learning objectives of a student at the beginning of a clerkship on the basis of feedback received in the preceding clerkship. An intake interview was considered a good way of initiating this process.       

As an outcome of this study, we implemented the following changes in our clerkships:

   •   All 4-week clerkships were lengthened to 6 weeks.

   •   A pilot study using the mini-CEX solely to provide feedback was successfully conducted in 2014 in the internal medicine clerkship (9 weeks). The mini- CEX forms were redesigned to create more space for feedback and now only contain a pass/fail judgement instead of grades. Subsequently, students reported receiving more feedback and being in favour of receiving only feedback instead of grades.^22^ In some clerkships, these feedback-only mini-CEXs have already been integrated. We plan to have them integrated in all clerkships in 2016.

   •   We have started providing an annual report on fail judgements for professional behaviour and clinical performance (of students) to individual hospitals, and we globally report the actions taken. This is to demonstrate to the faculty that their judgements are taken seriously, to show that there is a remediation trajectory and a follow up for students who fail the clinical performance and/or professional behaviour evaluations, and to show that some of the students need quite a long time for remedial work. This solution helped us in maintaining the confidentiality of student information (which is a requirement of our medical school) while still feeding the actions of the medical schools and the student performance back to the faculty. The general feedback in the report does not provide information on individual cases. However, we are aware that this is not a satisfactory solution to the particular problem mentioned by the faculty. We aim to keep looking for a solution to this problem.

We think that being attentive to the faculty’s predicament of the dual role of teacher and assessor is important. Although we have not implemented a concrete solution to this problem, we think that a possible way of helping could be to arrange community meetings for faculty (teacher communities) in which they can discuss their difficulties in conducting WBAs and share ideas and expertise. This could foster a sense of emotional support and confidence in their own judgements.^23^^, ^^24^

### Limitations of this study

We do not claim to have captured all the possible difficulties faculty could encounter in mini-CEXs and WBAs, but we think we have been able to identify the difficulties faced in our context. Our findings therefore require support from studies in different contexts. Furthermore, we did not use theoretical saturation as a guideline for data collection, but completed all the scheduled discussion meetings and included all the data in our analysis. This was because our meetings also aimed to collect information on the experiences of faculty to improve our clerkship programme and to provide faculty with a platform to express their difficulties. We did notice that after about 6 meetings, no new data were generated, which does suggest saturation.

## Conclusions

Our study highlights that faculties perceived difficulties with WBAs. Common difficulties included the number of encounters observed per student, the use of grades for the mini-CEX, the timing of the assessment, the combination of the role of teacher and assessor and perceived barriers to failing students. Faculty suggested longer clerkships in order to be able to have a higher number of encounters. They realised that they needed to be responsible for the timing of the assessment themselves. They also considered that they and the students would benefit from the mini-CEX to a greater extent if it provided only feedback instead of grades. They asked for forward feeding of previous evaluations of student’s competence and feedback on the progress of a student after a fail judgement. Our study suggests that WBAs need periodic review in order to address the difficulties faculty experience when using WBAs for evaluation and in order to provide feedback to ensure proper and effective use. A critical look at how WBAs are carried out is essential to optimise workplace learning.

### Conflict of Interest

The authors declare that they have no conflict of interest.
